# Supplementary material for: DOCK8 Mutation in Patient with Juvenile Idiopathic Arthritis and Sjögren’s Syndrome
Source: Int J Mol Sci. 2024 Feb 13;25(4):2259. doi: 10.3390/ijms25042259 (PMC10888949; doi:10.3390/ijms25042259)
Supplement: Supplementary file 1 [file ijms-25-02259-s001.zip › ijms-2848318-supplementary.pdf]

Supplementary Materials:

Table S1. Result of examination of basic lymphocyte subpopulations (2023).

Cells Subpopulation Phenotype	Absolute Values (thousands/mL)	Healthy Range	Percentage Values	Healthy Range
CD45	2,295		96,43%	
CD3	1,964	1,428 – 1,785	82,55%	60,00 – 75,00%
CD19	0,290	0,142 – 0,404	12,19%	6,00 – 17,00%
NK	0,072	0,285 – 0,554	3,03%	12,00 – 22,30%
NKT	0,112	0,023 – 0,119	4,73%	1,00 – 5,00%
CD4/CD3	1,125	0,795 – 0,974	57,28%	40,50 – 49,60%
CD8/CD3	0,512	0,567 – 0,785	26,07%	28,90 – 40,00%
CD4:CD8	2,19	0,8 – 2,5	2,19%	0,8 – 2,5
CD3/CD25	0,132		6,72%	
CD25 among CD3	0,010		7,6%	
CD19/CD25	0,002		0,87%	
CD25 among CD19	0,011		4%	
CD4/CD25	0,049		4,38%	
CD25 among CD4	0,075		6,74%	
CD4/CD25 (high)	0,039		3,51%	
CD25 (high) among CD4	0,063		5,31%	
CD4/PD1	0,638		56,76%	
CD8/PD1	0,137		26,75%	
**Cells subpopulation phenotype**	**Absolute values (thousands/mL)**	**Healthy range**	**Percentage values**	**Healthy range**
CD19/PD1	0,034		12,05%	

CD—Complex of differentiation; PD1—Program death factor—1.

Table S2. Clinical features of DOCK8 deficiency.

Main Symptoms	Atopic Diseases	Autoimmune Manifestations
recurrent respiratory infections	eczema	autoimmune cytopenias* hemolytic anemia
persistent viral cutaneous infections*	food and environmental allergies	systemic lupus erythomatosus
viral infection<br>bacterial infections<br>other	asthma*<br>eosinophilic esophagitis<br>allergic rhinitis	uveitis<br>arthritis*<br>vasculitis
failure of long-term vaccination responses	↑ IgE levels, eosinophilia	inflammatory bowel disease
increased risk of cancer*<br>↓NK cells*	Anaphylaxis (dru allergy*)	CUN vasculitis<br>glomerulonephritis

*—symptoms in the presented patient.

Methodology:

The whole exome sequencing library was prepared using SureSelect XT Library preparation Kit (from Agilent). The sample was sequenced with Illumina technology on NovaSeq6000 sequencer with 2 × 100 bp reads. The obtained QC value was 92.89% for Q30. Demultiplexing of the sequencing reads was performed with Illumina bcl2fast (2.19). Adapter was trimmed with Skewer version 0.2.9 [18]. The reads were aligned to GRCh37/hg19 reference sequence using BWA-MEM. Read duplicates were removed using Picard 2.18.2 (http://broadinstitute.github.io/picard/). Variant call was performed with GATK v4.0.3.0 HaplotypeCaller [19] and FreeBayes (v1.2.02-g29c4002). Variants have been annotated with databases: (i) VEP97 [20]: annotations Sift, Polyphen2, (ii) dbNSFPv4.0 [21] annotations: MutationAssessor, MutationTaster, DANN, FATHMM, (iii) ESP6500, (iv) GnomAD, (v) dbSNP, (vi) ClinVar and (vii) 1,000 Genomes. CNV analysis was performed using XHM [22].
